# Elements of Physiological Physics

**Published:** 1885-06

**Authors:** 


**Elements of Physiological Physics.** An Outline of the Elementary Facts, Principles and Methods of Physics and their application in Physiology. By J. M. Robertson, M. A., M. B , Assistant to the Professor of Physiology in the University of Glasgow. Illustrated with 219 Engravings on Wood. Philadelphia: H. C. Lea's Son and Co. 1884.

This is one of the valuable series of "Manuals for Studies," issued by Lea. The physician will find it both instructive and interesting. The chapters on electricity and magnetism, and their application in physiology and medicine, is a clear explanation of what by most practitioners is little understood. The elementary facts and principles of physics will, we hope, soon be a necessary part of the general knowledge demanded of the matriculates of our medical colleges. Those who have not had the opportunity to study such subjects are often now at a loss, when physics and chemistry are constantly appealed to, in working out physiological problems.

